# An Intelligent Surveillance Platform for Large Metropolitan Areas with Dense Sensor Deployment

**DOI:** 10.3390/s130607414

**Published:** 2013-06-07

**Authors:** Jorge Fernández, Lorena Calavia, Carlos Baladrón, Javier M. Aguiar, Belén Carro, Antonio Sánchez-Esguevillas, Jesus A. Alonso-López, Zeev Smilansky

**Affiliations:** 1 Dpto. TSyCeIT, ETSIT, Universidad de Valladolid, Paseo de Belén 15, Valladolid 47011, Spain; E-Mails: lcaldom@ribera.tel.uva.es (L.C.); cbalzor@ribera.tel.uva.es (C.B.); javagu@tel.uva.es (J.M.A.); belcar@tel.uva.es (B.C.); antsan@tel.uva.es (A.S.-E.); 2 Alvarion Spain SL, Parque Tecnológico de Boecillo, Edificio CEEI, 3.15, Valladolid 47151, Spain; E-Mail: jesus.alonso@alvarion.com; 3 Emza Visual Sense Ltd., 3 Hayozma st., Kfar Sava 44422, Israel; E-Mail: zeev@emza-vs.com

**Keywords:** smart visual sensors, surveillance, intelligent detection, security

## Abstract

This paper presents an intelligent surveillance platform based on the usage of large numbers of inexpensive sensors designed and developed inside the European Eureka Celtic project HuSIMS. With the aim of maximizing the number of deployable units while keeping monetary and resource/bandwidth costs at a minimum, the surveillance platform is based on the usage of inexpensive visual sensors which apply efficient motion detection and tracking algorithms to transform the video signal in a set of motion parameters. In order to automate the analysis of the myriad of data streams generated by the visual sensors, the platform's control center includes an alarm detection engine which comprises three components applying three different Artificial Intelligence strategies in parallel. These strategies are generic, domain-independent approaches which are able to operate in several domains (traffic surveillance, vandalism prevention, perimeter security, *etc.*). The architecture is completed with a versatile communication network which facilitates data collection from the visual sensors and alarm and video stream distribution towards the emergency teams. The resulting surveillance system is extremely suitable for its deployment in metropolitan areas, smart cities, and large facilities, mainly because cheap visual sensors and autonomous alarm detection facilitate dense sensor network deployments for wide and detailed coverage.

## 1. State of the Art in Intelligent Video Surveillance Systems

The concern for personal safety and security in public places is rising everywhere. Sales of video surveillance systems are expected to grow at a Compound Annual Growth Rate (CAGR) of 14.33% over the period 2011–2015 [1]. Research on video surveillance systems is therefore a hot topic, with many different areas being addressed at different levels. Some examples of typically very active areas are the cameras and visual sensors [2–4], the infrastructure to provide a network of sensors [5–8] and the field of intelligent video analytics [9,10].

New generations of visual sensors are constantly being explored. Some new sensors are enhanced with movement tracking capabilities based on Bayesian models [2], with effective background subtraction that enables later automatic video analysis [4] and with new planning tools that allow to configure their optimal sensing range, field of view and orientation in order to minimize the cost of the network of sensors [3].

Deploying dense networks of sensors is challenging because of power and bandwidth limitations. Both issues are due to the fact that the network of sensors need to multiplex hundreds of video streams in real-time, and in the uplink, while most of the network equipment is rather optimized to support more traffic in the downlink. To deal with this problem, several solutions have been proposed. An energy efficient image transportation strategy based on motion detection has been designed in [6] to tackle both the power limitations and the bandwidth limitation issues since it reduces the amount of frames to be transmitted. The limitations of 802.11 DCF MAC protocol specially in multi-hops scenarios has been addressed in [7] by designing a Time-Synchronized Application level MAC protocol (TSAM) capable of operating on top of existing 802.11 protocols; in addition, it can be used with off-the-shelf hardware and is capable of improving throughput and providing bounded delay. Another interesting approach to the performance of the network of sensors proposes a new schema for fair resource allocation operating at the application layer [8]. This system identifies critical network-wide resources and allocates them to the application level messaging units (called events) as opposed to regular flow of frames. In general, the idea is to provide some kind of scheduling capabilities to get some fairness and prioritization that complement a contention-based technology like 802.11.

Video analytics, which targets the autonomous understanding of events occurring in a monitored scene, is one of the main research trends in video surveillance systems. The idea is to have intelligent systems that are not only able to record video that can be later used as forensic evidence of crime or accidents but to help to avoid crime, terrorism and accidents in a proactive fashion [10]. Much of the research efforts in this field focuses on automatic tracking of objects and persons in motion [2,9]. However, video analytics systems process potentially sensitive information when using person tracking, behavior analysis or person identification [11].

Some companies are delivering sophisticated platforms to the market. A relevant player like IBM has presented a smart surveillance system which provides not only the capability to automatically monitor a scene but also the capability to manage the surveillance data, perform event based retrieval, receive real time event alerts and extract long term statistical patterns of activity [12].

Testing tools like ETISEO [13], a system for automatic testing of video surveillance systems, are already available as well as benchmarks [14] with realistic datasets that include actions by non-actor subjects.

Video surveillance systems are mainly intended to be used in big open public areas, such as municipalities, major events (such as Olympic Games [15], popular marathons) or in critical infrastructure protection [5] but they can also be applied in eHealth systems (e.g., in surveillance for fall detection of elderly people [16]). There are many examples of this trend, such as the 3,000 cameras of the Lower Manhattan Security Initiative [17] (aimed at picking up activities such as package delivery, and completed with car plate recognition, radiation detectors and mobile roadblocks) or London's “ring of steel” [18].

However, while the current trend is to continue deploying dense and wide area video surveillance sensor networks in order to provide all-time, all-location security, there are concerns that these systems are currently not reaching the promised levels, which has resulted in an increasing criticism across the society and media [19,20]. Therefore, it is becoming apparent that while this kind of systems has a huge potential, it is currently being underutilized, mainly because there is an overabundance and overflow of data which does not directly translates into information: video streams are properly captured, but intelligent analysis algorithms are not taking full advantage from them, and it is not feasible to have human operators watching them in real time. In the end, many of the videos are used for obtaining evidence after the crime has happened, but not for prevention or emergency management.

The focus of European Eureka Celtic Human Situation Monitoring System (HuSIMS) project is in two spots: first, in the video analytics systems to improve intelligent event detection; and second, in employing cheap intelligent sensors which reduce the amount of raw data sent to analysis to facilitate deployment of hundreds of thousands of units to public bodies, but also the implementation of private surveillance networks in smaller areas.

The visual sensors used in the project do not send in a regular basis video streaming but XML files containing perceived movements that are then processed by a powerful backend application in order to identify potential alarm situations. To the best of our knowledge, there is so far no attempt to build a system that combines the utilization of three different search engines with complementary approaches in the analysis of the visual sensors output. Our approach combines statistical analysis in the pattern revealing search engine, semantic web technology in the semantic search engine and relies on a rules-based system for the fusion of events and detected alarms. The utilization of text-based information in the analysis enables HuSIMS to be gentler with privacy matters.

After this summary of the state of the art, Section 2 presents the new requirements for surveillance systems and their overall system design; Section 3 deals with the intelligent visual sensors employed; Section 4 describes the network components to send the information to the Monitoring and Control System, including the intelligent alarm detection engines which will be described in Section 5; Section 6 presents two use cases; Section 7 compares the global HuSIMS system against the other solutions. Finally, Section 8 summarizes the conclusions of this work.

## 2. Vision, Principles and Architecture

### 2.1. New Surveillance Systems Requirements

HuSIMS intends to provide an intelligent video surveillance system for deployments in wide urban areas. The HuSIMS approach tries to differentiate from the main trend in current video surveillance systems that use High Definition cameras that required lots of bandwidth to transmit the video to control nodes. Instead of that, HuSIMS employs low-cost analytic visual sensors that are able to track objects in motion and send low-weight XML files instead of heavy video streaming to a backend application. Of course, this reduces the amount of required bandwidth and therefore the cost of the required network. This low cost feature will enable large and dense deployments in municipalities as part of the future Smart Cities, can be a good solution for the coverage of crowed scenarios like concerts and sport events, and facilitates the adoption and deployment of the system by other smaller private initiatives to secure critical private facilities [21].

HuSIMS' main target is to provide real-time alerts for *irregular activity* in both indoors and outdoors scenarios. The latency of sending XML files is much lower than that of sending video streaming in a regular basis. The files will be processed by the backend application that includes three alarm detection engines working in parallel in order to bring rich alarm detection.

HuSIMS defines as *irregular activity* an activity that is important to identify and that can actually be identified. For example a parameter violation situation in which a wrong object is detected in a wrong place at a wrong time and/or moving in a wrong way: this may translate, depending on the domain, into a car accident in which a vehicle is out of its way and has invaded the sidewalk, an individual breaking a security perimeter, or a crowd running away from a fire. How these situations are detected by the different intelligent engines will be detailed in Section 5.

The system will transmit video only during an alarm situation. When a situation has been confirmed as alarm state, the system operators and the first responders will be able to request live video streaming from the alarmed place. The same approach is applied to video storage, where only short periods of a few minutes will be stored—those related to confirmed alarm situations and that will be specifically requested to the visual sensors upon alarm detection.

The fact that the system is using low resolution visual sensors provides an additional advantage regarding governance and politics: the provided image does not usually allow recognition of people's faces, which makes the system more respectful of privacy. This enables HuSIMS to provide a novel and compelling trade-off between privacy and enhanced security in a public space.

Summarizing, the key points of HuSIMS are:

- To minimize the processing and intelligence required in the visual sensors and therefore its cost. The visual sensors used in HuSIMS are intended to be simple, low resolution ones with a limited processing capability which makes impossible the utilization of advance techniques to recognize faces or objects. The processing made at the visual sensors is limited to objects/persons detection and movement tracking.
- To minimize the amount of data to be transferred in the network of sensors. It the visual sensors are not expected to provide the main processing then the intelligence has to be transferred to a control center where the processing will take place. However, in such a case, the required bandwidth in the network would be huge, particularly in very dense networks of visual sensors. In HuSIMS, the visual sensors send to the processing node only the parameters resulting of the movements' tracking concerning the objects in the observed scene. The video signal will still be available to the human operators that ask to manually analyze a given scene but this transmission is done only on demand, this wouldn't be the default situation.
- Alarm detection based on the objects parameters. Since the video signal does not progress to the control center and the visual sensors don't make on their own the alarm detection, the processing made in the control center is based on the objects parameters, e.g., size, direction or speed.
- Distribution of alarms and relevant data in real time. The results of the intelligent analysis will progress using a network infrastructure that will include the public authorities, security corps and first responders. This network will be used to transmit the alarms notification, their location, and scripts for first assistance depending on the type of alarm detected to the human operators in real time. The alarm subscribers could be in motion when approaching the alarm place and can request on demand video signal using client apps in their smartphones, laptops and tablets.
- To use highly flexible connectivity solutions. The network infrastructure used in HuSIMS combines several technologies like WiFi (802.11), WiMAX (802.16) and PLC (Power Line Communications) that enable the system to connect dense networks of sensors in both Local Area Networks (LAN) and in Metropolitan Area Networks (MAN). HuSIMS provides a Self-Organized Network (SON) whose nodes have self-configuration and self-healing capabilities.

### 2.2. HuSIMS System Design

**Figure 1.**
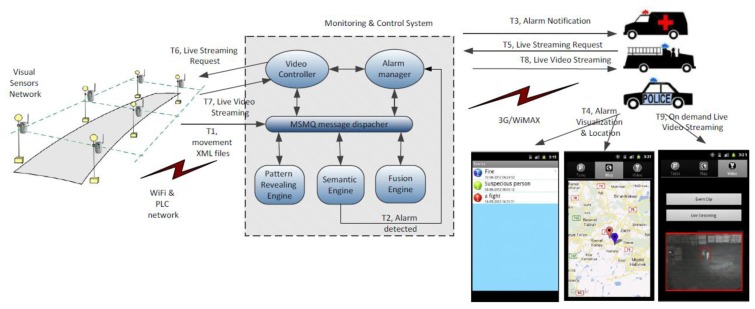
HuSIMS: alarm detection, notification and video streaming request upon alarm detection.

HuSIMS operates at three functional levels: movement tracking, alarm detection and alarm notification, as seen in Figure 1 (T1-T4). A dense network of visual sensors is on charge of the movement detection and tracking. Each visual sensor that detects movement in the scene that is watching sends a XML file with the object's motion parameters to the Monitoring and Control System (MCS). The visual sensors are able to filter shadows, weather conditions and many uninteresting noise-movements like those of the trees' leaves. The network of visual sensors is based on a combination of WiFi (802.11) and Power Line Communications (PLC) for covering both indoors and outdoors scenarios.

The XML files sent from the visual sensors are processed in the MCS by three different search engines that work in parallel detecting alarm situations at the MCS. Each search engine uses a different search strategy. The Pattern Revealing Engine aims at learning recurrent patterns in the motion parameters and raises alarms when the detected pattern does not correspond to one of the learned ones. The Semantic Engine translates to formal semantic formats the content of the XML files and uses ontologies for understanding what is happening on the scene and subsequent alarm detection. The Fusion Engine is a rule-based system that is able to detect alarm situations and is also able to correlate alarms detected by the other two search engines with the appropriate rules. When an alarm is detected the First Responders (police patrols, ambulances, firemen vehicles) are notified and receive in a mobile application information about the detected alarm, a script with tasks to be done to tackle the alarm situation and the alarm's location.

When an alarm state has been determined and notified, the first responders can request a live streaming session using their mobile application. The relevant visual sensor will receive the request and provide the streaming session that will be properly encoded by the Video Controller component at the MCS (see Figure 1, T5-T9).

## 3. Intelligent Visual Sensors

### 3.1. State of the Art in Security Cameras

The natural modality of obtaining information on the world around us is visual—we obtain more than 90% of information about the world surrounding us with our eyes, and about half of the human brain is busy with interpretation of this information. Even small animals, birds and insects can easily interpret the visual world surrounding them—this with a fraction of the computational power of an ordinary computer. Unfortunately, current artificial vision systems are usually bulky, expensive, and instead of having cognitive capabilities are often limited to image recording.

The concept of using motion cameras for security dates back to the 1940s, with the first commercial analogue video surveillance systems becoming available in the 1970s [22]. Initially, a person was required to continually monitor the video stream. Introduction of video recorders and later video multiplexing allowed greater flexibility in viewing, storing, and retrieving the (analog) video. Digital, or IP video cameras were introduced in the 1990s. Only recently have the sales of IP cameras outgrown those of analog cameras in the surveillance world. A recent, thorough and historical review can be found in [23].

It was quickly recognized the human observation of surveillance streams is ineffective [24]. As computers became prevalent, automated analysis of video became a topic of interest [25]. Such algorithms often used simple frame-to-frame differencing and thresholding, so called Video Motion Detection (VMD). Among the first applications were traffic monitoring [26] and intruder detection [27]. Automated analysis of video sequences has captured academic interest, which has earned the name “video analytics”, has been unsuccessful. While the size of the video surveillance market is above $14 billion, the size of the “analytics” market barely reaches $100 million, less than 1% of the totalmarket; numerous companies that were active in this field disappeared, and none of them reached sales of over $10 million. The selling hype, together with under-performance, so discouraged users that today “analytics” has become synonymous with “non-functionality” [28] and the term has practically disappeared.

Automated analysis of video streams usually relied on a separated architecture, where the cameras relay live video to a central video server facility. Such servers often attempt to perform costly operations such as edge detection, tracking, object recognition and even gesture recognition, applying complex computational and mathematical operations [29,30]. There are very few products where the analysis is tightly coupled with the acquisition camera. These are sometimes termed “edge cameras”, although this includes cameras that record their video on-board, rather than remotely. Another approach meant to reduce the reliance of video surveillance on expensive infrastructure is the hybrid surveillance sensor, where a camera is in deep sleep mode, being awakened by a low-power sensor (e.g., a Passive IR, PIR). Again, this is simply a recording or transmitting instrument, with no analytical capabilities. Finally, while exaggerated claims are being made as to what automated analysis of video can achieve (“identifying suspicious people”, “left luggage at a busy subway station”), such products have yet to penetrate the market and gain commercial traction. Thus, there are no current solutions to real-time alerting of irregular events in metropolitan areas, particularly such that require low-cost, low-complexity infrastructure.

In the research community, the topic of “smart cameras” and “embedded smart cameras” has gained considerable interest (cf. reviews in [23,31,32]). Some recent studies considered tracking performed by a network of embedded smart cameras [33,34] and specific hardware architectures [35]. Recent designs for low-energy surveillance systems include a hybrid low-resolution stereo “sensor” coupled with higher resolution color camera [36], development of light-weight algorithms for embedded smart cameras [32,37], and smart camera networks for agricultural applications [38].

### 3.2. HuSIMS Intelligent Sensor

**Figure 2.**
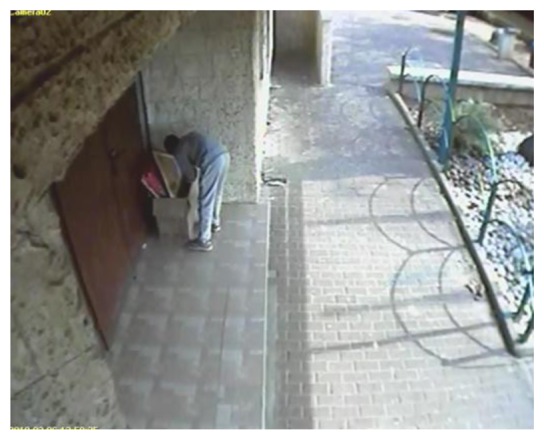
Example of an irregular event with a strong visual signature: single person loitering near the community center main entrance door (resulted in a foiled arson event, courtesy: security department of the city of Nes Ziyona, Israel).

The intelligent visual sensor developed for HuSIMS is novel in several respects. First, it normally produces no video output—only a digital (XML) description of the activity observed in its monitored scene. Second, it aims to reduce drastically the device's profile—in terms of size, cost, required bandwidth, power consumption, and installation complexity, while keeping high end performance in varying weather and illumination conditions. In contrast with some of the publications mentioned above, the HuSIMS visual sensor relies on standard components and architecture, with a CIF or VGA CMOS sensor (mobile phone type) and ARM9 processor running proprietary, low computational cost algorithms, at 10–15 frames per second (FPS). These features make it a true visual sensor, and not a camera. All this allows the sensors to be densely deployed, each one monitoring a limited city area (e.g., a road junction, a pub entrance, or a bus stop). Finally, the visual sensor is meant to serve the HuSIMS system, where *irregular events with a strong visual signature* (Figure 2) are detected at real time and relevant verification video is streamed to policemen or other security professionals.

The AVS architecture is meant to maximize the data obtained from each pixel, to allow reduction of the visual sensor resolution and processor power. The algorithms are built in three layers: pixel layer, segment layer and object/motion layer. A main innovation is the design of the low-level, or pixel layer. This part is the most computationally intensive, and effort has been taken to make it as efficient as possible. Our approach calls for simplification of the computational blocks, contrasting with traditional image processing approaches, which tend to consider the image acquisition device as a measurement tool and the processing of its data as an implementation of *rigorous mathematical tools* such as Gaussian edge detectors, Fourier analysis, and convolutions. Such operations require floating point arithmetic and complex architecture or application specific hardware such as DSP or FPGA which can be power hungry. Our approach to the pixel level processing is inspired by nature's visual processing architecture, which utilizes a large number of simple analog receptors, each sensitive to a particular aspect of vision: color, contrast, motion, direction, spatial resolution, *etc.*

Since we focus on static sensors that stare at a fixed scene, each pixel must determine whether, at a given frame, the light intensity impinging on it is regular, or irregular, with respect to the historical intensities it experienced over a prescribed interval (several seconds to a few minutes). The classical implementation of such an operator requires digitally storing the historical intensity levels and computing the relevant statistics. However this implies significant storage and computational resources. Our approach captures pixel historical behavior using a lower and an upper adaptive threshold. These thresholds define the envelope of expected intensities of a “regular” incoming pixel signal.

While this approach in which new pixel values are compared to historical statistics is already known, its light-weight, extremely efficient implementation (making use of the two threshold approach and an elementary, almost analog update schema) is in fact a sensible innovation in the field. This algorithm uses only integer arithmetic, incrementations/decrementations, comparisons and table lookups, making this sensor able to analyze real-time video on a low-performance ARM9 processor.

**Figure 3.**
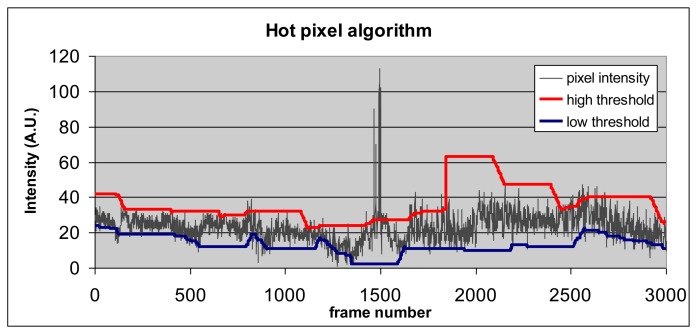
Hot pixel algorithm, showing the high and low thresholds and their adaptive behavior as a function of pixel intensity profile.

An example of thresholds' behavior as a function of pixel intensity profile is shown in Figure 3. When pixel intensity exceeds the value of the high threshold (e.g., frame 1500 in Figure 3) or falls below the low threshold, it becomes “hot”.

The following stage, segmentation, identifies connected components of hot pixels. A basic merit figure of any surveillance system is the minimal detectible object size, in pixels. For example, for a minimal object size of 8 pixels, the expectation of a random occurrence of eight specific pixels being simultaneously hot is p8 where p is the random expectation of a pixel to be hot. Setting p = 1/100 and assuming VGA format and 30 FPS, a single event of 2 × 4 random pixels (anywhere in the frame) will occur once in 100^8^ = 10^16^ events. This is equivalent to 30 FPS × 31,536,000 seconds/year × 307,200 VGA pixels × 34.5 years. Thus if the pixels are tuned to sensitivity of 1:100 frames, good detection sensitivity can be achieved with low inherent false alarms and a small detectable form factor, maximizing the use of each pixel and avoiding the necessity for a large number of pixels, reducing further the power consumption.

Analysis and experience show that a p factor of 0.01, while could be initially regarded as simplistic, is very general, allowing the (static) sensors to operate well in a large variety of applications, including intruder detection, safe city or home security. This can be compared to the natural process of adaptive retinal sensitivity, which is a universal process that performs similarly indoors and outdoors, night and day, in urban, vegetated or desert regions of the globe. Reducing this value (say to 0.001) will reduce drastically the sensitivity of the system, while increasing it (say to 0.1) will result in numerous false alarms.

### 3.3. Innovations and Comparison

Table 1 below summarizes the innovation of the HuSIMS visual sensor compared to available systems. We compare the HuSIMS visual sensor with three of the current market leading systems. The first is the VideoIQ system that includes analytics and recording onboard the edge unit. The second is Mobotix, a manufacturer of high-end megapixel cameras and video management systems. One key feature of Mobotix is that the video can be transmitted directly to storage. The third comparable is Axis, the market leader in IP surveillance cameras.

## 4. Network Components

HuSIMS requires two different networks from a functional point of view: a network of visual sensors for data collection and an alarm distribution network for the notification to first responders of the confirmed alarms.

Both are private networks. It is becoming increasingly common to physically separate emergency application networks from carrier networks—both cellular and landline. When emergencies arise, carrier and operator networks fast become overloaded and mission-critical emergency applications can cease operating. In a privately-owned and operated safe city network, the city can control traffic, define priorities and make sure that the network is always available for the critical applications when they are needed. Finally, private networks cost less to operate than leased networks.

While VPNs (Virtual Private Networks) could have been a viable solution (even presenting some advantages like increased flexibility), there are some concerns regarding their application in real environments, mainly the problems that may arise due to sharing bandwidth with other applications which might result in delays or information loss. For critical security applications, this is sometimes unacceptable. Ultimately, the choice between real and virtual private networks will bring different features into the system.

### 4.1. Outdoor/ Indoor Network for Alarm Collection

The data collection network will connect the sensors to the MCS application. Its main functionality will be to transport the XML files sent by the visual sensors to the MCS where they will be processed. When constructing the visual sensors network, cost-effective and easily deployable technologies are used. Houses and Buildings infrastructure includes coaxial cables, power lines and phone line cables. Reusing this infrastructure for surveillance allows a massive fast deployment of the visual sensors in indoor scenarios.

In outdoor scenarios, Wireless networks can be deployed quickly and are very flexible. Wireless technologies like WiFi (802.11) and WiMAX (802.16) make it possible to add and place a very dense amount of cameras and visual sensors in locations previously inaccessible, and offer Quality of Service (QoS) management, high-capacity, high-availability, built-in data encryption mechanisms and low latency connectivity essential for real-time high-resolution video streaming overlarge geographic areas.

However wireless equipment is in general designed to support most of the traffic in the Down Link (DL). In video-surveillance applications most of the traffic goes in the Up Link (UL). Therefore the wireless equipment used in HuSIMS in the alarm collection network was re-designed to support a flexible balancing of the traffic. One of the main targets of HuSIMS is to be a cost-effective system that may be deployed in large and heterogeneous areas. The deployment of Wireless + Wired solutions (e.g., WiFi-enabled visual sensors linked to the power line network using Power Line Communications—PLC technology) allows wireless, fast and low cost deployment with “zero cost” using existing wiring and providing a double linked network to guarantee QoS, capacity and availability.

The new generation of wireless equipment developed for the project includes new Self-Organization Networks (SON) features like self-configuration and self-healing. Self-configuration allows the quick deployment of sensors following a ‘plug-and-play’ paradigm and enables to download new configuration parameters and new software versions. This is achieved using TR-069 enabled CPEs and Alvarion's Automatic Configuration Server called StarACS.

Self-healing helps to reduce the impact of failure in a given network element allowing the sensors that were connected to the failing node to find connectivity via adjacent cells and enabling quick addition of new sensors and replacement of the damaged ones. HuSIMS network has a flexible architecture that enables the visual sensors to be in constant communication with the MCS. Rather than using mesh networks whose performance quickly degrades in multi-hop scenarios, the system employs point to multi point wireless access nodes based on 802.11n protocol. Regarding network planning, the visual sensors will always be able to reach more than one access node in order to provide redundant paths for the visual sensors to reach the MCS. On the other hand, in indoors scenarios, two technologies like WiFi and PLC communications will be used one as backup infrastructure of the other in order to get always-on connectivity to the MCS.

Self-healing features will allow quick addition of new sensors and quick withdrawal and replacement of damaged ones. Self-healing helps to reduce the impact of failure in a given network element allowing the sensors that were connected to the failing node to find connectivity via adjacent cells.

### 4.2. Outdoor Wireless Network for Alarm Distribution

The alarms distribution network will connect the MCS application with the first responders (police patrols or emergency vehicles).

While the sensor network emphasizes the low-bandwidth required, in alert or emergency situations, the bandwidth requirements increase significantly due to the real time distribution of the video signal to the emergency teams. This network needs to be based on a technology that supports broadband and mobility for enabling mobile users to receive the bandwidth demanding video necessary to have a clear idea of what is happening on-site. A network based on WiMAX (802.16e) thanks to its QoS management features has been chosen and enhanced with optimized video transmission capabilities for that purpose.

The video optimization feature allows that video streaming sessions using MPEG-4 codec get a special treatment that ensures the quality of the video transmission in situations of air resources shortage. In MPEG-4 codec the video frames can be classified into Group of Pictures (GOP) in which three different types of frames can be found. Key frames, or I frames that are coded independently; P frames, which include delta updates of I frames; and B frames, which are bi-directional frames. Losing I frames impacts the entire GOP. P frames are second in priority and B frames would have the lowest priority. We use three different queues to classify each type of frame. Using this approach to classify the video frames, when not all video packets can be transmitted (e.g., air resources shortage), we will prioritize I packets and drop P/B packets as necessary.

## 5. Monitoring and Control System

### 5.1. State of the Art in Intelligent Alarm Detection

Nowadays, intelligent surveillance is a field of research that is constantly growing. New intelligent cameras, sensors, multi-camera environments, *etc.* require the development of new technologies to take advantage of the raw data retrieved by these components and transform it in useful, high level information for the operators. For this, several data processing alternatives are being developed, such as scene understanding, face/plate/object recognition, or alarm detection.

The application of complex machine vision algorithms is one of the main trends for video surveillance [39–42]. However, they normally have strong requirements on computing power, so either the sensor itself packs a powerful processing unit, making it expensive, or the high definition video signal is sent to a central processing unit, a solution with high bandwidth consumption (especially in a scenario with large numbers of cameras). Those limitations are solved by systems that employ lighter paradigms to process the image, which normally imply reducing the video stream to a set of second level parameters (such as object movement [43]. These approaches present all the advantages of being lighter, but usually left out much information in the image (such as color or shape of objects) when reducing the video stream to parameters. Therefore, they require advanced analysis tools to maximize the high level information that can be extracted and its subsequent interpretation.

One of the solutions is the probabilistic approach. Systems such as the ones presented in [44,45] use Bayesian network based solutions. Bayes' Theorem is very useful to determinate the probabilities of an alarm by using the relations between all the variables in a specific situation. Other authors [46] prefer more complex probabilistic approaches like Hidden Markov Models (HMM) (typically employed in many pattern recognition domains), to extract unknown but meaningful parameters from raw data provided by the cameras. Other techniques, used in pattern recognition too, are the Dynamic Time Warping (DTW) [47] and Longest Common Subsequence (LCSS) [48]. Those mechanisms compare groups of variables to find similarities among them, and they are successfully employed, for instance, to group similar trajectories in surveillance scenes [49,50].

The presented Bayesian network solution and similarity based methods use the explicit information provided by the system to detect alarms. The HMM-based systems go beyond. They extract new implicit information, hidden in the raw data provided by the cameras and sensors. Some deductive techniques use Neural Networks [51,52] or Clustering Algorithms [53] to classify behaviors and contexts but they are normally resource-greedy and data processing is slow.

On the other hand, along the last 10 years have witnessed the development of the Semantic knowledge technologies, a new approach for formally representing and processing knowledge (using knowledge models known as ontologies) which was first applied in the World Wide Web (giving birth to the Semantic Web, or Web 3.0), but which was quickly extended to other fields, including intelligent surveillance, with good results [54,55]. Semantic technologies offer several advantages, like easy interoperability among heterogeneous systems and easy adaptation to different application domains by replacing ontologies.

Modern surveillance systems normally comprise big numbers of cameras and sensors. Typically, video signals from these sensors have been treated independently, but there are many cases in which their outputs can be combined in order to get a better understanding of what is happening, and even for detecting events which might slip undetected through the analysis of a single scene. In order to take advantage of the increased situational awareness that emerges from the combined interpretation of several sensors outputs, data fusion techniques have been also applied [56,57] with good results.

The HuSIMS alarm detector aims at combining all the advantages of the different techniques exposed by the application of three different analysis engines in parallel. The result is system which can cover a dense network of cameras and sensors, reliably detecting anomalous situations.

### 5.2. HuSIMS Monitor and Control System

**Figure 4.**
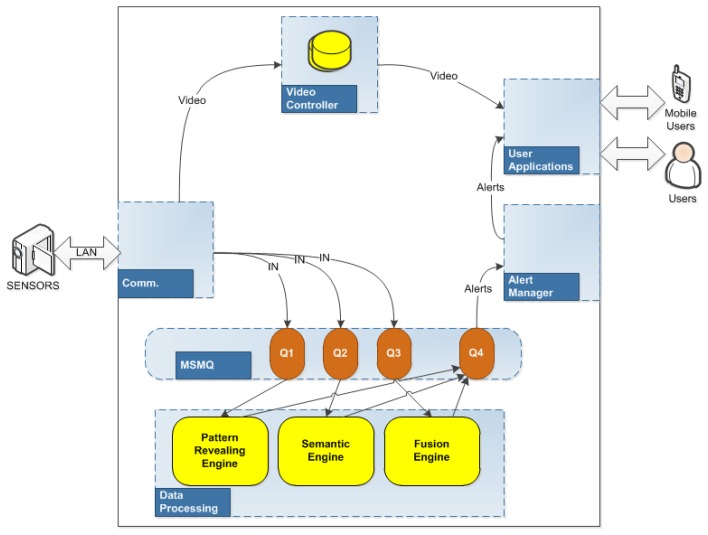
MCS architecture.

The objective of the HuSIMS' Monitor & Control System (MCS) module is to control the data flow through the different parts of the system. In order to implement that function, the MCS system includes separate modules for communication, data analysis, and user applications—as Figure 4 shows. The MCS communication module receives the information from the visual sensors through the Meshed Sensory Network (MSN) and forwards the raw data to the system core, the Data Processing Module, using a MSMQ (Microsoft Message Queuing) technology. This data processing module is composed by three complementary engines which follow different data processing strategies which are able to distinguish between normal and abnormal situations (patterns) at the monitored area:

- The Pattern Revealing Engine converts the moving object data in Key Performance Indicators (KPI) information and learns their typical patters. An alarm is then raised when the pattern starts to drift out from a normal one.
- The Semantic Engine performs the semantic characterization of the information sent from the MSN. The system is based on the interpretation of the watched scene in terms of the motion parameters of the objects, giving a semantic meaning to them, and using Semantic Web technologies. A semantic reasoner process identifies when an emergency situation is developing.
- Finally, the Fusion Engine is an advanced rule engine dealing with the problem of how to fuse data from multiple sources in order to make a more accurate estimation of the environment. Users can add their own mathematical or semantic functions in order to create new rules. These rules are used to create a behavior later. In a behavior, a case is defined by functions, real data fields and rules. Output of a running behavior is the expected result set of scenery.

When the Data Processing detects an alert the Alert Manager receives the details and forwards them to the user through the User Applications. If the user needs to watch the situation, this application also requests the video of the scene to the MSN using the Video Controller Module.

### 5.3. Pattern Revealing Engine

The Pattern Revealing Engine is based on a family of algorithms [58] for automated modeling and characterizing of any sequence of KPI values in the operational dataset. The model is a data structure that specifies the conditional probability of any KPI value, given past or current values of other KPIs in the sequence (called the context). The model is coded by a network of trees that represents all the patterns in the data that are statistically significant. A specific context-Based Forecasting (C-B4) algorithm is employed to optimize the size and the statistical efficiency of the model, depending on the triggered application [59,60].

Once the model is constructed, it captures all the significant dynamics and dependencies in the data for each KPI. The Pattern Revealing Engine detects anomalies in data sequences, based on patterns rather than the data values, while maintaining relevant and readily interpretable results. Whereas traditional performance-management techniques are usually limited to using KPI control limits to identify problems, HuSIMS Pattern Revealing Engine can detect pattern's anomalies before KPIs exceed their control limits.

The HuSIMS' algorithm is as follows:

a) Algorithm for pattern generation: each new data received can be stored as a possible extended pattern of a previously detected pattern, if indeed this extension is justified by certain information-theoretic measures. The way the pattern is being constructed, as an additional branch in an existing tree, is actually a patented algorithm.
b) Pattern grading: each new pattern is associated with given likelihood grade based upon information theory metrics (as the amount of new information contained in the new pattern). For HuSIMS needs, the grading algorithm is separated from the pattern generation algorithm to enable fast and near real-time performance.
c) Algorithm for decision: this module can identify if the new pattern, with its grade, is indeed a new pattern, or in fact it is similar to some previously detected patterns. This algorithm is crucial for the monitoring application, since it provides the ability to detect new patterns in a “true-true” needed confidence level.
d) Clustering algorithm: builds clusters of patterns to facilitate handing.
  - All those algorithms include sub-algorithms, to ensure a high level of confidence in the received results. For example, these algorithms can be used to find anomalies in KPI correlations, even when the KPIs themselves behave normally, pattern matching provides measures of the similarity (or difference) between data sequences that can be used to compare and classify them. This can be used to classify errors in operational datasets.
  - To aggregate different KPIs to support pattern generation and identification.
  - For root cause analyses for faults and errors in the system.

After a training period the C-B4 system is capable of detecting, for example, a normal state of movement direction inside a specific area in the monitored area. If there is a change in these KPI resulting from an abnormal movement direction in that area, an alert is automatically. It is worth noting that no rules are set a priori, the specific normal situation is learnt automatically by the algorithm.

### 5.4. Semantic Engine

The Semantic Engine is based on projecting the sensed data into a knowledge model representing a human interpretation of the data domain. Specifically, the Semantic Engine developed for HuSIMS [61] operates with only with the simple object motion parameters provided by the visual sensors while other semantic systems use advanced image processing techniques like object and shape recognition in order to identify meaning.

**Figure 5.**
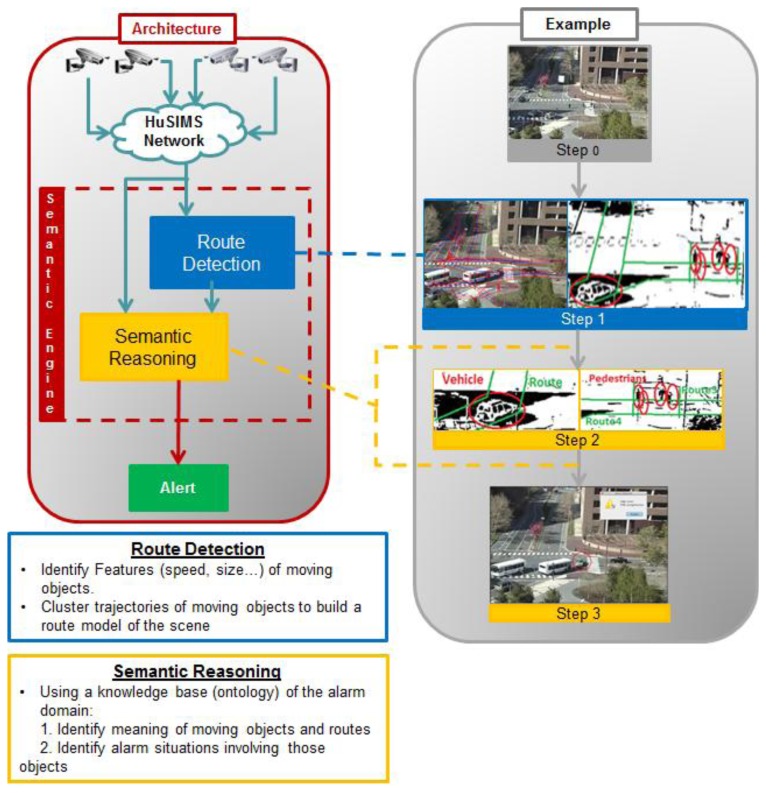
Architecture of Semantic Engine.

Figure 5 represents the Semantic Engine's internal architecture. There are two main modules called “Route Detection” and “Semantic Reasoning”.

The Semantic Engine receives from the MSN a file with the data received by each visual sensor and forwards it to the “Route Detection” module. This module processes every frame and using a set of algorithms, it determines the routes, *i.e.*, the zones of the scene where objects habitually move in, by clustering their trajectories. After Step 0 representing image capturing, Step 1 in Figure 5 shows an example with the result of the Route Detection process. The right side of the image shows how the pedestrian and vehicles detected in the scene are the inputs to the Route Detection module to determine the scene trajectories (in green). In the left side, the trajectories detected in the real scene after training are presented. Route Detection is performed only when the system is in training mode, and when finished, this model is fed to a second block, called Semantic Reasoning, so as to be inserted in the ontology and used during the operation mode.

When the system is in operation mode, the information extracted from the frames received is sent to the Semantic Reasoning too. This block uses Java and Jena (an extension of Java which implements a semantic framework) to process the previous information and populate the ontology with the semantic information about the individuals appearing in the image. Finally, the semantic reasoner (a Generic Rule Reasoner is used for this HuSIMS implementation), which is the core of the Semantic Reasoning block, processes the ontology, recently populated with the new data, to infer properties about the objects in the image and label them according with the type of object identified. In Figure 5, Step 2 shows how the labels Vehicle or Pedestrian are given to moving objects, and the detected routes discriminated with tags like Road or Sidewalk. Then, with this new inferred information, the Semantic Reasoning specifically identifies if an alarm situation is going on (see Step 3 in Figure 5). If it is the case, an appropriate Alarm is sent towards the MCS.

### 5.5. Fusion Engine

The main purpose of Fusion Engine is to analyze the information collected by several visual sensors and runs pre-existing fusion algorithms, mining the data with the purpose of identifying surveillance anomalies automatically.

The Fusion Engine executes a generic, user-friendly information fusion process via a database using rules, which can be defined at run time. There are three different layers at which rules can be defined: Behavior Development Level, Rule Development Level and Atomic Development Level. At the Atomic level, a super-user can introduce new mathematical/atomic functions in the platform, at run time using Java. These atomic functions can be used to implement new rules at the second layer that can act as simple behavior templates. Then, these templates can be composed together at the top level in order for the user to implement specific behaviors.

The Fusion Engine consists of a central database and three coordinated and simultaneously working engines:

- Generic Data Identification Engine (GDIE).
- Generic Data Fusion Engine (GDFE).
- Generic Data Output Engine (GDOE).

**Figure 6.**
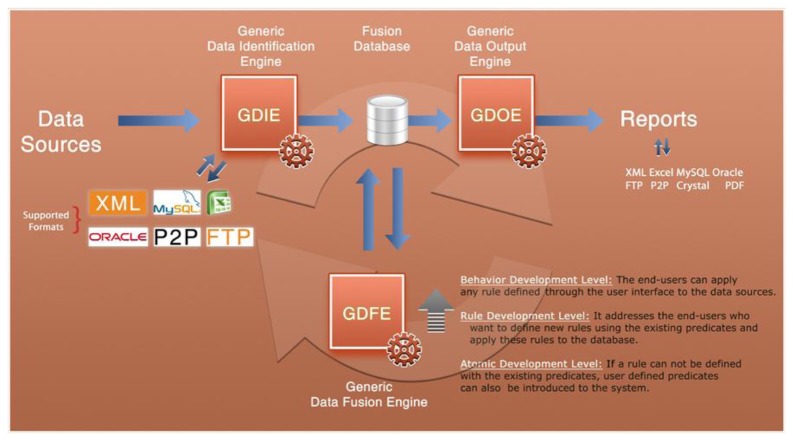
General structure of Generic Data Fusion Engine.

The relationship between these components and the different composition levels are shown in Figure 6.

The first of these components is a GDIE, which allows to connect different data sources (XML files, Excel files, information stored in a MySQL database, *etc.*) and sensors and to collect information from them. After data verification, format definition and mapping the fields into MySQL, the data is inserted into the central database.

The GDFE associates the gathered data in central database with each other and fuses them as rule-based.

The last component, the GDOE exports and reports the fused data in several formats. It can generate not only dynamic reports but also custom reports by using predesigned report templates. On the other hand, if the fusion results include location data, results can also be shown on a visual map. In HuSIMS project, Fusion Engine templates are sent using Web Services to a Mobile Event Manager that shows the event information to the user in his terminal, for instance using Google Maps, Bing Maps or Apple Maps.

**Figure 7.**
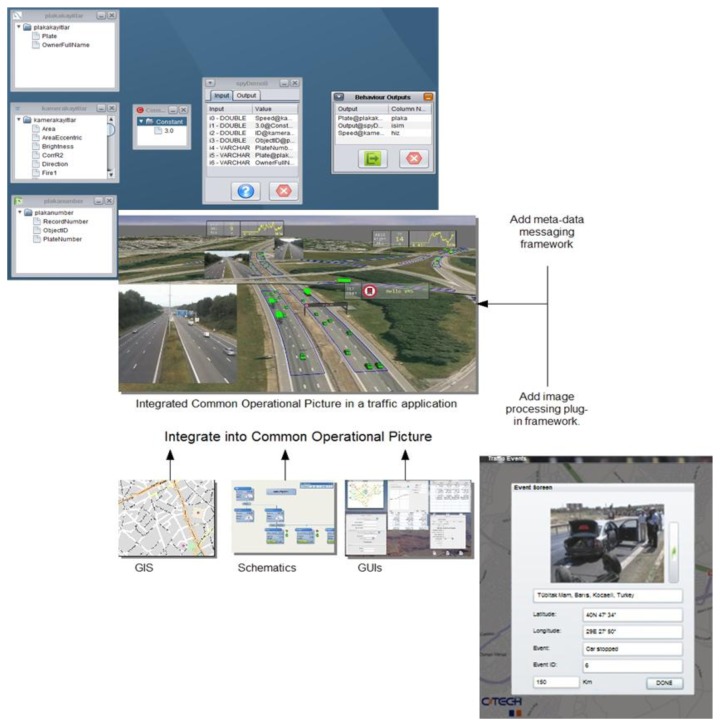
Fusion Engine operation.

Figure 7 shows how the Fusion Engine throws an event using the information sent by several visual sensors. The left side of the image presents the information provided by the different sensors and sources (as represented in the engine's user interface). In the middle one, all that information reported by different sensors is merged in the same scene and processed to identify the alarm situation show at the right side of the image.

## 6. Alarm Detection Use Cases

This section presents several use cases where the operation of the system is shown. The system has been tested with video data processed using the visual sensors software.

### 6.1. Traffic Management

This use case shows how the HuSIMS system is employed in a traffic management scenario and is capable of detecting vehicles driving in the wrong direction.

**Figure 8.**
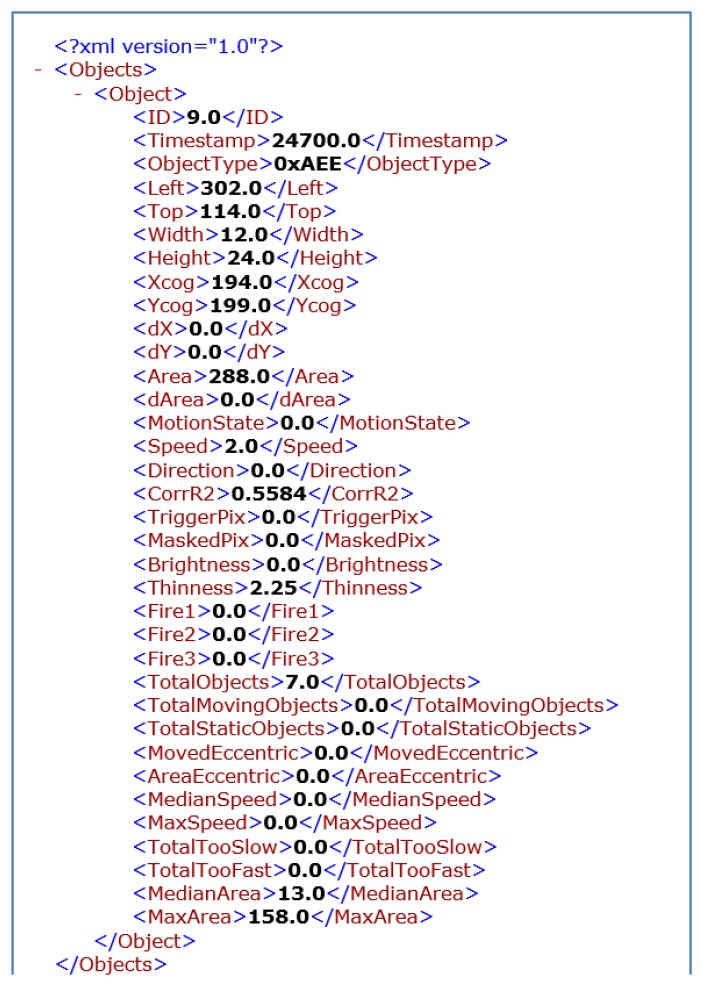
Example of XML file.

First, the real video is processed by one of the visual sensors that provides information about the movement objects. Whenever a moving object is detected in a frame, an XML file (See Figure 8) is sent towards the MCS reporting the main features of all the objects detected. These means that reports are not sent on a continuous basis, but only when meaningful information is available. Frame rates are also configurable, and the use case has been tested for instance with frame rates of 10 FPS. For each object the visual sensor reports width, height, position (x, y), area, speed, direction of the movement, brightness and thinness, and maximum historical values for area, width, height, speed and brightness. In addition, each object has an id to allow tracking along the scene.

During the training process, the engines learn the correct/normal values for the scene, each of them using their own abstractions.

**Figure 9.**
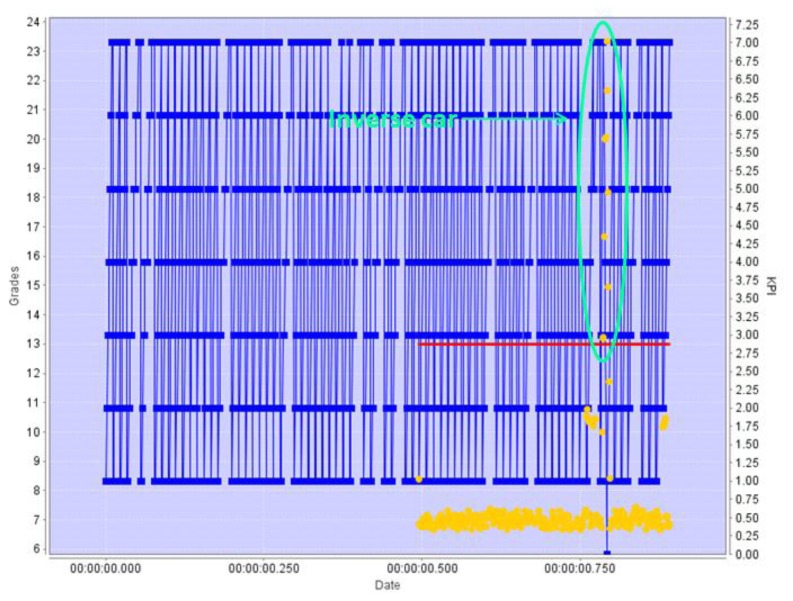
A car driving in the wrong direction, a change in the pattern of direction of movement is detected, even before car completed the full change of direction. The grades referred to the directional KPI indicates a new pattern of the direction KPI.

Figure 9 represents the behavior of the Pattern Revealing Engine in this scenario, with the x-axis representing time, and the *y*-axis representing at the same time the values of the position KPI and the grading of patterns.

First, during the training period the Pattern Revealing Engine identifies all the significant patterns in the position, speed, and direction KPIs for the objects reported, building a scene-specific pattern model. The values reported are represented in the Figure 9 as blue points. Once the system enters the operation mode, each newly detected pattern is given a score (in grades), which are represented in Figure 9 with a yellow dot. Those grades indicate how much the new pattern is similar to the previously learned “normal” set of patterns. The grade received is a measure of how different is the pattern being analyzed when compared of the normal patterns: if the grade is low, the current pattern detected in the KPI evolution is quite similar to one of the learnt ones. The red line is a threshold line: if the score (yellow dot) is above this line, the difference of the current pattern with the learnt ones is meaningful, and an alert is issued.

Figure 9 is an example of detection by the C-B4 grading algorithm. During the times before the red line appears in the image the Pattern Revealing engine is working in training mode, receiving information from a camera watching a one-direction highway where cars go south. When it enters the operation mode, the figure shows yellow dots for each graded pattern, which for the most part represent cars travelling in the right direction. However, at some point, the grades obtained start to surpass the threshold: looking at the blue line it is easy to see that at this point there is a change in the trend exhibited by the calculated KPI. This change in the trend is caused by a car that was detected travelling in the wrong direction.

The Semantic Engine follows a different approach to deal with the same case. First, a human ontology engineer designs a traffic management ontology, which states that “road” objects may have a preferred direction, and defining that detecting a car travelling in the opposite direction should be identified as an alarm state. This ontology is loaded in the Semantic Engine.

Then the Semantic Engine has to learn a route model for each specific sensor. To build the model for this use case, the Engine is set up in training mode, which in the end learns the usual routes of the objects and the direction of the movement in those routes, and according to the parameters “area” and “speed” of the objects populating them (as specified in the ontology), they are labeled as a “road” or a “sidewalk”.

**Figure 10.**
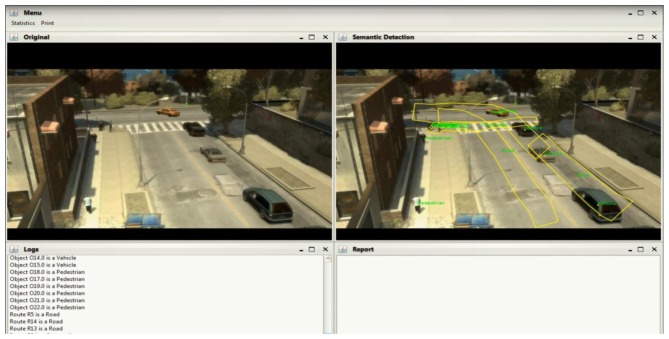
Example of a Semantic Engine's learning process.

Figure 10 shows an example of how this route model is built inside the Semantic Engine during the training mode. The left side of the picture presents the original scene, while it right side also includes the labels assigned to routes (road, crosswalk, *etc.*) and objects (pedestrian or vehicle). While it is not shown in the image, the system stores many internal parameters about the routes, such as the direction of movement and the type of objects usually moving inside it.

When it enters the operation mode, the Semantic Engine no longer learns routes, but assigns labels to the moving objects depending on their parameters. Route R5 (the lane at the left side of the road, where cars move downwards), for example, was identified as “road” and the direction of the vehicles that move through it was 5 (using the hours in a clock as a reference). When a vehicle with a direction of 11, that vehicle is identified as moving in the opposite direction and an alarm is thrown.

**Figure 11.**
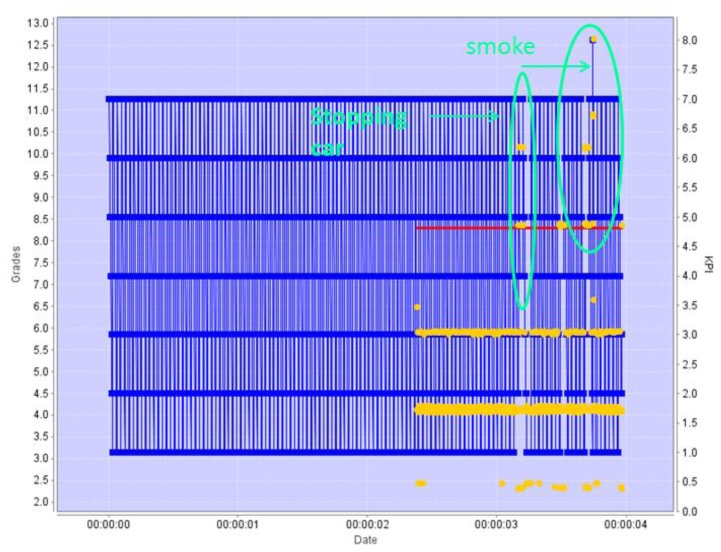
Stopping car—the normal movement KPI were recognized as a stable pattern. Once a change in the velocity data pattern was recognized—an alert is issued.

Both engines will work similarly for detecting cars abnormally stopped in the road. Figure 11 shows the KPI and grading scheme for the Pattern Revealing Engine, also presenting a clear change in the KPI trend for two cars stopped in the middle of the road, which is correctly detected by the grading algorithm. The second car is actually facing a breakdown which causes smoke to come out of the engine. The movement pattern of the “smoke” object is so different from the learnt ones that the value of the KPI comes out of the positional range observed up to that point: the smoke is moving in a zone of the image where no object has been moving before.

The Semantic Engine will also detect that a vehicle is stopped in a zone where there should not be any stopped object: on a pedestrian crossing. This abnormal behavior has to be coded inside the ontology.

It is worth mentioning that for a car stopping before a red traffic light or a stop signal, for instance, no alarm is issued in any of the cases. The Pattern Revealing engine would have learnt positional patterns that represent cars stopping at those points in the image, and the Semantic Engine would have labeled that part of the image as a “road before a traffic light” because objects labeled as “cars” would have previously stopped in that area of the scene.

### 6.2. Vandalism Detection and Crowd Control

The HuSIMS system can be easily deployed in many different scenarios, such as vandalism detection and crowd control. When applied to these domains, the system will identify abnormal behavior of people, and the movement parameters monitored by the cameras will be enriched with microphones reporting noise levels. In the case of a fire in a pub, the cameras will report abnormal values of the brightness parameters for the flames, and some minutes after abnormal motion parameters, because the people in panic trying to escape. During training, the Semantic Engine will have identified forbidden areas and places where people move freely, which will be characterized in the crowd control ontology together with parameters such as average speed. First, a “flames” alarm will be issued to the MCS when an object with an abnormal brightness parameter is detected in a place where a light is not identified, and afterwards—when people starts moving at an unusually high speed in unusual places as they try to escape—they are labeled as people running. As the ontology specifies that many persons running represent a crowd in panic, and an alarm of this type is issued.

The Pattern Revealing Engine will act similarly, identifying brightness and movement patterns extremely different from those learnt during training, and therefore identifying and abnormal event.

Another scenario where the system may result useful is vandalism detection. In this example, a person punches another during a fight in a subway station. It should be noted that identifying this kind of abnormal situation solely on the basis of the action *per se* is extremely difficult. Even an extremely focused human operator will have trouble discriminating this action from two friends shaking hands, for instance, specifically taking into account the low resolution of the sensors. However, the HuSIMS system relies on the assumption that vandalism actions are usually accompanied by erratic or strange behavior before and after the event. In the case of the fight, it is extremely unlikely that the attacker will simply hit another person and continue walking normally. Instead, he will probably leave running.

Even more, HuSIMS supports the usage of additional, non-visual features and parameters in order to help in detecting this kind of visually-confusing situations. For instance, the previously mentioned noise detector could help identifying screams and shouts related to a fight; and in the case it is happening inside a subway station, a detector of the presence of a train will help discriminating if the noise is coming from a train entering the station or it is due to an abnormal/alarm situation.

Under these circumstances, the Pattern Revealing Engine and the Semantic Engine will both detect that there has been and abnormal event. The Semantic Engine will even specify a fight alarm.

### 6.3. Data Fusion and Engine Collaboration

The Fusion Engine will help in putting together all the multimodal information provided by the different sensors of the architecture and the alarms issued by the engines. For instance in the case of the fight described in the previous subsection, the Fusion Engine is capable of tracking the running suspect across the scenes as reported by the different sensors, effectively identifying the route followed. It will identify that the object moving at an abnormal speed which is sequentially reported by different sensors is the same individual, and will report his position in real time to the authorities.

But the Fusion Engine will also help in reducing the number of false positives, by combining the output of the other two engines. For instance, in the case that there is a sensor camera for surveillance in a street where there is a shop with a window display, during training it might happen that all pedestrians either enter the shop, or continue walking along the sidewalk. However, during the operation mode, a pedestrian stops in front of the window display in order to view some of the items in which he is interested. The Pattern Revealing Engine may understand this action as an abnormal event, since no other pedestrian has stopped there before, issuing an alarm which is false in this case. On the other hand, the Semantic Engine understands that it is not an alarm that a pedestrian is stopped, as long as he is on a sidewalk, and therefore does not issue an alarm. The Fusion Engine will, in this case, combine both outputs (using a set of rules defined by the operator) to identify that it is a false alarm (and differentiating it from the many situations in which the Pattern Revealing Engine will simply identify alarms that are not covered by the ontology in the Semantic Engine); therefore, in this case, the alarm identified by the Pattern Revealing Engine will not be progressed to the MCS.

## 7. System Comparison

HuSIMS presents several distinct features when compared to other available solutions in the literature and in the market. Most notably, the usage of cheap visual sensors, the conversion of low-resolution video streams into a set of object motion parameters making unnecessary the streaming of video in a regular basis, AI reasoning over those motion parameters for autonomous alarm detection, and generic, domain-agnostic approaches to scene processing (which effectively qualify the system for multi-domain operation—traffic surveillance, vandalism, perimeter security, *etc.*), represent a very particular philosophy which stands HuSIMS apart from alternatives. Table 2 summarizes a top-level feature comparison of HuSIMS and other reported initiatives following very different approaches.

## 8. Conclusions

In this work, the HuSIMS video surveillance platform has been presented. It has been designed with wide area, dense deployments in mind, using inexpensive sensors with low resource/bandwidth requirements in order to facilitate deployment and management of thousands of sensors, and generic, multi-domain reasoning and alarm detection engines. This makes HuSIMS a perfect candidate for integrated surveillance systems in smart cities and big facilities where it is necessary to count with large numbers of cameras to provide full coverage of the entire area.

In addition, the three alarm detection engines present innovative solutions on their own: the Pattern Revealing Engine enjoys the ability of learning meaning-agnostic patterns in the motion parameters sent by the cameras; the ontology domain knowledge model employed by the Semantic Engine allows to understand what is happening in the scene; and the data Fusion Engine is capable of extracting information from the combination of outputs. The parallel operation of three different strategies provides a wide range of different alarm detection features, because the system is capable of providing meaningful and rich information about the different known situations, as represented by the ontologies in the semantic engine, and at the same time detect abnormal behaviors which have not been explicitly specified in a knowledge model. As a result, the system is robust (alarms are detected according to an expert knowledge model) and dynamic/flexible (unexpected but abnormal situations are also detected) at the same time.

## Figures and Tables

**Table 1.** Comparison of current video surveillance systems with the HuSIMS visual sensors.

	**VideoIQ**	**Mobotix**	**Axis**	**HuSIMS Visual Sensors**
**Aim**	Analyze and record video in place, allow remote access	Record video and transmit directly to storage	Record video	Describe a dynamic scene with thin XML data
**Front end**	Video camera, analytics and storage	Day/night megapixel camera + microphone	Camera	Visual sensor, interprets the scene and transmits thin XML description
**Back end**	Video servers performing image analysis	Storage device/ analytics channel	Video recorder/ storage/analytics channel	Statistical engines analyzing and correlating activity data from hundreds of visual sensors
**Analytics**	Intruder detection	None	None	Automatic detection of anomalous events, per sensor, per time of day
**Infrastructure**	Fiber-optics for trans	mitting live video, high powe	r consumption	Low wireless bandwidth, low power consumption

**Table 2.** HuSIMS feature comparison.

	**HuSIMS**	**Current State of the Art**	**ADVISOR [62]s**	**ARGOS [63]**	**DETER [64]**	**AVITRACK [65]**
Objective	Alarm Detection	Recording video	Send warnings to human operators	Boat traffic monitoring	Alarm detection	Monitor and recognize activities
Resolution	Low	High	384 × 288 pixels	320 × 240 pixels	High	720 × 576
Bandwidth	Low	High	Ethernet IP Multicast	Local PC connection. (No specified connection with the control center)	Coaxial cable	1 Gb Ethernet
Storage	Rarely, only important video streams	Always	Yes (video + annotations)	Yes	Yes	Yes
Privacy	Gentle	Aggressive	Aggressive	Gentle	Gentle	Aggressive
Cost	Low	High	High	High	High	High
Type of Data Analyzed	Motion parameters	Video Signals	Video Signals	Video Signal	Video Signal	Video Signal
Domain	Multi-domain		Centered on metro stations	Maritime traffic detections (designed for Venice)	Vehicle and people detection	Airport Security
